# Creating an Integrated System of Data and Statistics on Household Income, Consumption, and Wealth

**DOI:** 10.23889/ijpds.v9i5.2912

**Published:** 2024-09-18

**Authors:** David Johnson, Mahsa Jessri

**Affiliations:** 1 National Academies of Sciences, Engineering and Medicine; 2 University of Wisconsin - Madison

The nation's disparate federal statistics makes it difficult to accurately measure income and wealth inequality and other aspects of economic well-being for the nation's households and families. This report provides the recommendations from a panel appointed by The Committee on National Statistics (CNSTAT) of the National Academies of Sciences, Engineering, and Medicine. The report reviews the major income, consumption, and wealth statistics currently produced by U.S. statistical agencies, provides guidance for modernizing and integrating the information to better inform policy and research, and builds on the current effort at CNSTAT to create a 21st Century Data Infrastructure to use blended data from multiple sources. In addition to providing guidance on the appropriate definitions of household, family, and individual income, consumption, and wealth, and variations in definitions that would be useful for particular purposes, the report provides suggestions for pathways forward using multiple data sources to construct a data infrastructure for income, consumption and wealth for households. The report highlights new statistics that help in measuring the disparities in income, consumption and wealth and the changes over time in both levels and inequalities, always stressing that the disparities occur both across socio-demographic characteristics and geography. The report highlights the potential for blending multiple data sources and modeling across data sources, including surveys, tax records, state and federal administrative records, and commercial data, and documents the legal and administrative barriers to creating integrated, high-quality estimates and a data infrastructure for researchers to evaluate the impacts of public policy.

